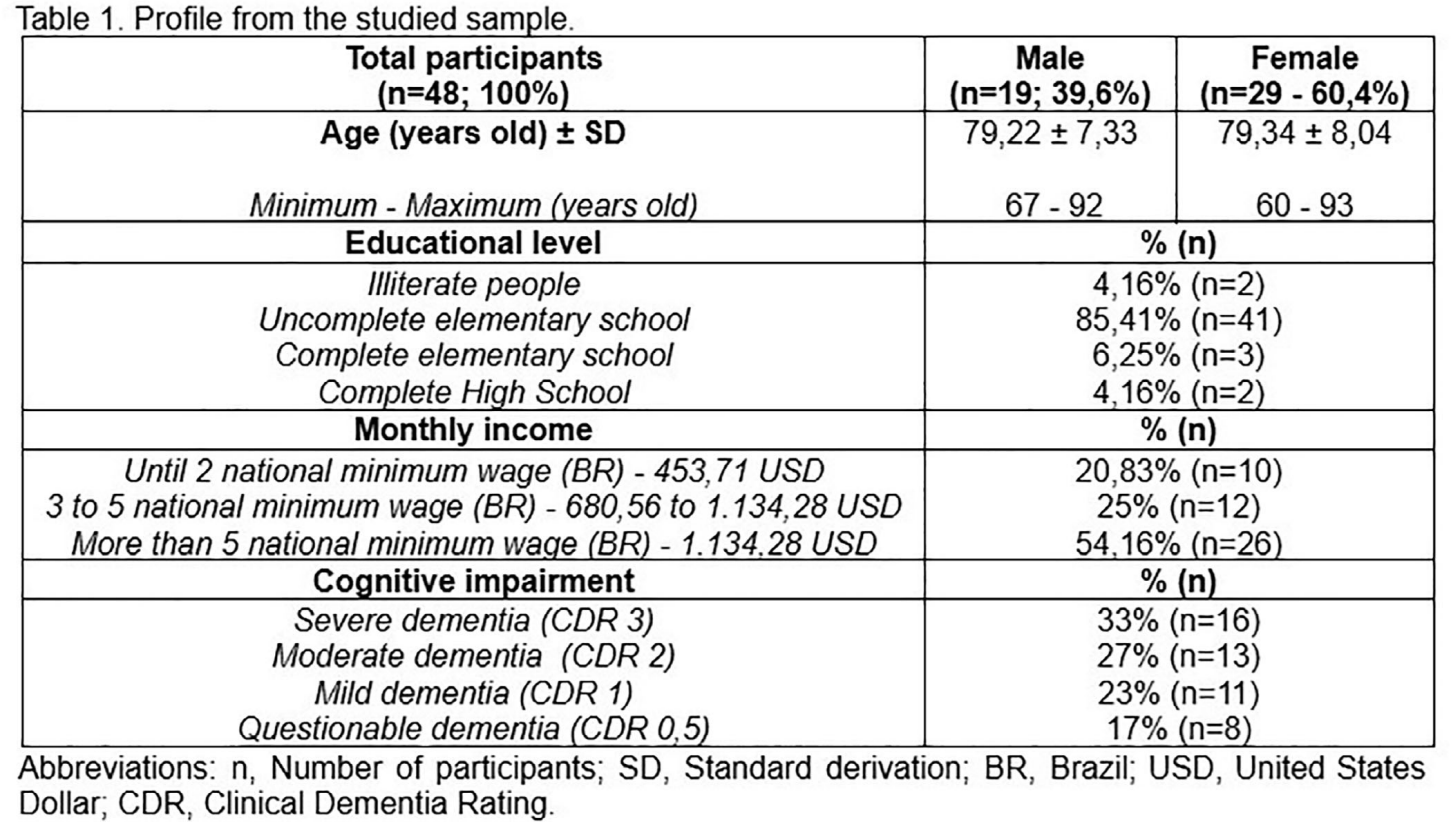# Cognitive and sociodemographic data of elderly individuals with Alzheimer's Disease attended by a family‐support service in a southern city of Brazil

**DOI:** 10.1002/alz70860_100887

**Published:** 2025-12-23

**Authors:** Fernando Sluchensci dos Santos, Renan Felipe Pereira Gonçalves, Roberta Fabbri, Renan Cassiano Ratis, Murilo Bastos, Fernanda Correa De Melo, Weber Claudio Nunes Franscisco Da Silva, Juliana Sartori Bonini

**Affiliations:** ^1^ State University of the Midwest, Guarapuava, Paraná, Brazil

## Abstract

**Background:**

Alzheimer's disease (AD) is the most common form of dementia worldwide, particularly among elderlies and women, whose risk increases with advancing age, lifestyle, and genetic background. Family history, education level, and income are factors that may influence the progression of AD. This study aimed to assess the cognitive and sociodemographic profiles of elderly individuals with AD receiving support from a family‐assistance service.

**Method:**

This observational study was performed in 2023 in the central region of Paraná state (Brazil) and was approved by the Research Ethics Committee of UNICENTRO, following the Declaration of Helsinki. Recruitment of elderly participants was carried out through the local public health system. Sociodemographic data were collected using specific questionnaires and semi‐structured interviews.

**Result:**

A total of 48 elderlies were recruited. The average age of participants was 79.28±7.68 years. Women represented 60.4% of the sample (*n* = 29). Most of individuals had not completed elementary school (*n* = 39; 81.25%). The monthly income exceeded five times the national minimum wage in 54.16% of families (*n* = 26), earning an estimated value of 1,134.28 USD. Severe cognitive impairment (CDR 3) was present in 33% of the sample (*n* = 16), moderate impairment (CDR 2) in 27% (*n* = 13), and mild impairment (CDR 1) in 23% (*n* = 11). Questionable dementia (CDR 0.5) was identified in 17% of participants (*n* = 8).

**Conclusion:**

Severe dementia staging (CDR 3) was observed in the largest portion of individuals evaluated in this study, which may be associated with the low education level and income of this population. Investing in research with an epidemiological focus is crucial for studying chronic diseases, including AD, because focuses on the potential for early intervention which significantly impacts on quality of life.